# Investigating the Thermal Stability of Omega Fatty Acid-Enriched Vegetable Oils

**DOI:** 10.3390/foods13182961

**Published:** 2024-09-19

**Authors:** Katalin Nagy, Bogdan-Cezar Iacob, Ede Bodoki, Radu Oprean

**Affiliations:** Analytical Chemistry Department, “Iuliu Hațieganu” University of Medicine and Pharmacy, 4, Louis Pasteur St., 400349 Cluj-Napoca, Romania; nagy.katalin@umfcluj.ro (K.N.); iacob.cezar@umfcluj.ro (B.-C.I.); roprean@umfcluj.ro (R.O.)

**Keywords:** thermal degradation, GC-MS, vegetable oils, fatty acid profile, omega-3 fatty acids, omega-6 fatty acids, trans fatty acids

## Abstract

This study investigates the thermal stability of omega fatty acid-enriched vegetable oils, focusing on their behavior under high-temperature conditions commonly encountered during frying. This research aims to evaluate changes in fatty acid composition, particularly the degradation of essential omega-3, -6, and -9 fatty acids, and the formation of harmful compounds such as trans fatty acids (TFAs). Various commercially available vegetable oils labeled as containing omega-3, omega-6, and omega-9, including refined sunflower, high-oleic sunflower, rapeseed, and blends, were analyzed under temperatures from 180 °C to 230 °C for varying durations. The fatty acid profiles were determined using gas chromatography–mass spectrometry (GC-MS). The results indicated a significant degradation of polyunsaturated fatty acids (PUFAs) and an increase in saturated fatty acids (SFAs) and TFAs with prolonged heating. The findings highlight the varying degrees of thermal stability among different oils, with high-oleic sunflower and blended oils exhibiting greater resistance to thermal degradation compared to conventional sunflower oils. This study underscores the importance of selecting oils with favorable fatty acid compositions for high-temperature cooking to minimize adverse health effects associated with degraded oil consumption. Furthermore, it provides insights into optimizing oil blends to enhance thermal stability and maintain nutritional quality, crucial for consumer health and food industry practices.

## 1. Introduction

Oils and fats are fundamental in human nutrition, with over 90% of global production used in food directly or as components of food products [1]. Dietary fats are necessary for the transport of fat-soluble vitamins and other health-promoting substances, making them vital for a balanced diet [2]. Moreover, oils also significantly influence global commerce and economic landscapes [3].

The human body is unable to synthesize essential fatty acids (EFAs), which include omega-6 (n-6) and omega-3 (n-3) fatty acids, thereby necessitating their intake through the diet. These EFAs and their longer-chain derivatives like arachidonic acid (C20:4 n-6, AA), eicosapentaenoic acid (C20:5 n-3, EPA), and docosahexaenoic acid (C22:6 n-3, DHA) are linked to various health benefits [4], such as reducing cardiovascular disease risks [5] and promoting a healthy inflammatory response [6]. The competition between omega-3 and omega-6 fatty acids for enzymatic processes [7] highlights the importance of a balanced intake of these fats for optimal health benefits [8,9]. Vegetable oils are primary sources of EFAs, with their fatty acid compositions varying across different types. Notably, oils such as flaxseed, rapeseed, and high-oleic sunflower oil are noted for their high omega-3 and omega-6 content. In recent years, the market of omega-3 and omega-6 enriched vegetable oils has evolved significantly, reflecting the growing awareness and preferences of health-conscious consumers. Fortified vegetable oils serve as both flavorful dressings for salads, lending a nuanced taste to dishes, and as versatile cooking media employed for frying.

Frying is a common cooking method that enhances the taste and texture of food but also leads to the formation of harmful compounds through thermal, oxidative, and hydrolytic reactions [10,11,12,13]. Primary oxidation products, like hydroperoxides, due to their poor stability at elevated temperatures, degrade rapidly into secondary products, both volatile and non-volatile, such as aldehydes, ketones, and alcohols [14,15]. The non-volatile degradation products hold significant importance in both chemical and nutritional contexts because they persist in the oil, become incorporated in the food, and are subsequently consumed [16]. Repeated frying, especially in the presence of food matrices, can generate toxic compounds like glycidol and malondialdehyde, both of which are harmful to human health [17,18,19]. Degraded oils not only produce harmful compounds but also negatively affect sensory qualities like aroma, color, and viscosity [10]. Simultaneously, the smoke point of the oil (the temperature at which the oil decomposes) decreases [20,21]. All of these factors impact both the functional and nutritional qualities of the frying oil. Cooking with degraded oil at high temperatures has various adverse effects on human health, including weight and growth suppression, increased liver and kidney issues, and damage to the thymus and epididymis [22]. Furthermore, consuming abused frying fat is difficult to digest and can result in diarrhea. Despite partial digestion, this fat is absorbed into the human body, raising toxicological concerns [23].

The oxidative stability of oils, crucial for maintaining their quality and health benefits, is influenced by their fatty acid composition, the presence of natural antioxidants or pro-oxidants, and environmental factors like light and humidity [1,13,24,25]. The stability of frying oils, both in terms of chemical composition and sensory attributes, also relies on the temperature [26] and duration of the deep-frying process [14]. Polyunsaturated fatty acids (PUFAs) are prone to degradation at high temperatures, leading to the formation of trans fatty acids (TFAs) and other harmful compounds [27,28,29,30,31,32]. The high intake of TFAs is associated with an elevated risk of cardiovascular diseases and insulin resistance [28,33,34,35,36,37] due to their negative impact on cholesterol levels [38] and their disruptive influence on fatty acid metabolism [39,40]. The global consumption of TFAs, despite a decrease over recent decades, remains high in several regions, emphasizing the need for the careful selection and use of cooking oils. Considering its negative impact on health, the World Health Organization (WHO) suggests minimizing TFA intake, ensuring it contributes no more than 1% of total daily energy intake [41]. Contrary to PUFAs, monounsaturated fatty acids (MUFAs), exemplified by oleic acid (C18:1c n-9, OA), exhibit a higher resistance to oxidation [42]. A prior investigation revealed that the oxidation rates for stearic acid (C18:0), OA, linoleic acid (C18:2 n-6, LA), and alpha-linolenic acid (C18:3 n-3, ALA) were in a ratio of 1:100:1200:2500 [43]. This heightened vulnerability leads to diminished oxidative stability during various stages of technological processing, storage, and usage.

In the current market, vegetable oils enriched with omega-3 and omega-6 fatty acids are receiving significant attention, offering consumers a diverse range of options to meet their nutritional needs and culinary preferences. To create a nutritious oil blend, various oils are typically mixed. For better stability during cooking, omega-3- and omega-6-enriched vegetable oils should have a balanced PUFA/MUFA ratio and include antioxidants [44,45,46,47]. The practice of blending omega-rich vegetable oils with standard refined plant-based oils is a modern technique adopted by leading brands [48].

Marketing strategies for omega-fortified vegetable oils are closely aligned with the expanding variety of new vegetable oils and the rising consumer demand for health-focused food products. Packaging is designed to be eye-catching and informative, highlighting the omega content and using phrases such as “heart-healthy” and “cholesterol-free”. The design often features green and other natural hues to subtly link the product with health and nature. Additionally, it is crucial to adhere to health claim regulations and ensure that product labeling is precise and supported by scientific evidence, and promotes ethical marketing. This is especially relevant in the context of the diverse regulatory landscape across the European Union, where regulations on the use of altered oils in deep-frying vary significantly [49]. Some countries have established specific guidelines to limit components like ALA [34], reflecting the importance of maintaining high standards in product quality and consumer safety in the marketing of omega-fortified vegetable oils. However, marketing omega-enriched oils also has drawbacks. Promoting these oils might cause an imbalance in dietary fat intake. Fortified foods can lead to excessive consumption of certain fatty acids, especially omega-6, which may upset the beneficial omega-3-to-omega-6 ratio and potentially cause health problems like inflammation and cardiovascular disease. Furthermore, the production and marketing of omega-fortified oils raise environmental concerns, such as increased demand for omega-3 from marine sources potentially leading to overfishing. The ethical issues concerning sustainability and the environmental impacts of sourcing are often overlooked in marketing these products.

Considering the intricate relationship between marketing strategies that highlight the health benefits of omega-enriched oils and the scientific challenges associated with their thermal stability, this paper aims to examine the thermal degradation of vegetable oils, assess the impacts of heating processes, and compute nutritional indices to better understand potential changes in their health effects. This analysis will provide crucial insights into how marketing strategies align with the scientific realities of using omega-enriched vegetable oils in everyday cooking practices.

## 2. Materials and Methods

### 2.1. Oil Samples and Materials

To provide a comprehensive analysis, a wide selection of commercially available vegetable oils from various brands, primarily sourced from Romania, were purchased from local supermarkets. The selection included oils marketed with labels indicating omega-3, omega-6, and omega-9 content. Alongside these specialized oils, conventional vegetable oils from the same brands were also analyzed. The range of samples included one refined rapeseed oil branded as omega-3 (BO3); two refined blends of sunflower and rapeseed oils labeled as omega-3, omega-6 (FO36), and omega-9 (FO9) from the same brand; a blend labeled with omega-3 (COO3) comprising sunflower, grapeseed, corn, flaxseed, and rice bran oils; and two high-oleic sunflower oils enriched with omega-9 (SO9, VOO9). Conventional oils from all five brands, including two varieties of extra virgin olive oils (CO, COIG) and four refined sunflower oils (B, F, S, VO), were also analyzed. An additional refined sunflower oil (A) and two types of extra virgin sunflower oils (BS, SS) were included as well. Comparisons were made between these specialized and traditional sunflower oils from the brands to ensure a thorough evaluation. The bold letters indicate the brand of the oils. Extra virgin olive oils were denoted as “EVOO”, extra virgin unrefined sunflower oils as “URS”, refined sunflower oils as “RS”, refined rapeseed oils as “RR”, and mixed oils as “MX”. As commercial products, the frying oils were without the addition of antioxidants and were used before their best-before date (Table 1).

Gas chromatography (GC)-grade hexane and a boron trifluoride–methanol solution (BF3-MeOH) for GC derivatization were purchased from Sigma-Aldrich (Burlington, VT, USA). A potassium hydroxide solution in methanol (NaOH-MeOH) was purchased from Merck (Darmstadt, Germany). The Supelco 37 Component FAME Mix certified reference material and internal standard (IS), methyl nonadecanoate, were purchased from Sigma Aldrich (Burlington, VT, USA) and used during the validation procedure.

### 2.2. Frying Treatment

Approximately 15 mg vegetable oil samples were weighed directly into standard glass tubes and heated at 180 °C, 200 °C, and 230 °C using an Ecocell 111 oven (BMT, Monroe, WA, USA). At 230 °C, the samples were taken at different time points: unheated (0 min), T30 (30 min), T60 (60 min), T180 (180 min), and T360 (360 min) after heat treatment. Samples were taken immediately after reaching the desired temperature and consequently were analyzed by gas chromatography–mass spectrometry (GC-MS).

### 2.3. Preparation of Fatty Acid Methyl Esters

The fatty acid composition of the samples was determined as fatty acid methyl esters (FAMEs). FAMEs were obtained by transesterification of oils with BF_3_·MeOH after alkaline hydrolysis. The vegetable oil sample was mixed with 1 mL 0.5 mol/L NaOH in methanol, and the mixture was saponified at 70 °C for 30 min. After cooling to room temperature, 2 mL 14% BF_3_·MeOH solution was added and the mixture was heated at 70 °C for 30 min to promote the formation of the methyl ester. Again, the mixture was cooled to room temperature, and 4 mL saturated saline solution was added to terminate the reaction, followed by the addition of 2 mL hexane. The solution was allowed to settle until two layers were formed, and the supernatant was collected and transferred into GC vials prior to analysis.

### 2.4. Gas Chromatography Analysis

FAMEs were analyzed by GC-MS using an Agilent 7890A gas chromatograph system coupled with a mass-selective detector (MSD) 5975 C inert XL EI (Santa Clara, CA, USA), operated in electron impact mode with an ionization energy of 70 eV. Separation was performed on a Zebron™ ZB-FAME by a Phenomenex Inc. GC capillary column (30 m × 0.25 mm I.D. × 0.2 µm film thickness) (Torrance, CA, USA). The injector port temperature was maintained at 250 °C and a splitless injection mode was chosen. Ultra-high-purity helium 6.0 was used as the carrier gas at a flow rate of 1 mL/min. The temperature program was as follows: the initial temperature was set as 100 °C, held for 2 min, and increased to 140 °C at a rate of 10 °C/min, further increased to 190 °C at a rate of 3 °C/min, and then at 30 °C/min up to a final temperature of 260 °C and held for 1 min. The MS transfer line was maintained at 280 °C and the ion source and quadrupole temperatures were set to 230 °C and 150 °C. The mass spectra were recorded over a range of 50 to 550 atomic mass units at 0.5 s/scan. The components were identified by comparison with their mass spectra with the spectrometer database of the Wiley275 and NIST Library and further confirmed with the Supelco 37 component FAME mixture. The quantification of individual fatty acids was conducted by the internal standard method and area normalization method.

### 2.5. Multivariate Data Analysis

Principal component analysis (PCA) was performed on the obtained fatty acid profiles of the analyzed vegetable oil samples after performing unit variance scaling and mean centering of all quantitative variables (K = 11, normalized percent content in unsaturated, mono- and polyunsaturated fatty acids C16:1, C18:1c (n-9), C18:1t (n-9), C20:1 (n-9), C18:2 (n-6), C18:3 (n-3), C16:0, C18:0, C20:0, C22:0, C24:0) using Simca version 17 software (Sartorius Stedim Data Analytics AB, Umeå, Sweden).

## 3. Results and Discussion

### 3.1. Fatty Acid Profile of Vegetable Oils

The present study focuses on the comprehensive analysis of fatty acid profiles in omega-enriched and commonly used vegetable oils, with a particular focus on investigating changes in the essential dietary fatty acid composition during the heating process. The GC-MS method was developed to analyze the fatty acid composition of the vegetable oils. A total of fifteen prevalent vegetable oils frequently utilized for frying were subjected to analysis to elucidate their respective fatty acid compositions pre- and post-heating, revealing noteworthy disparities.

The method validation showed strong performance across several key parameters. Selectivity was confirmed with a 37 Component FAME Mix, ensuring clear peak resolution and no interferences in the vegetable oils. Linearity was excellent for all 11 FAMEs, with regression coefficients above 0.99. The precision met the acceptance criteria, with %RSD values under 0.25% for retention times and under 13% for peak areas. Mean recovery was 96.74%, with minimal bias, indicating high accuracy. The method’s sensitivity was demonstrated by the LOD and LOQ values, and the methylation and extraction process showed recovery rates of 82.41–109.11% with low RSDs, confirming its reliability.

The initial analysis of unheated oils revealed considerable variations in fatty acid composition (Table 2). Refined sunflower oil, for instance, exhibited prominent levels of LA (48.07–54.05%), OA (26.18–40.17%), and saturated palmitic acid (C16:0) (5.89–12.38%), having the highest PUFA and saturated fatty acid (SFA) contents among the oils studied. Conversely, it displayed the lowest MUFA content, resulting in a diminished percentage of omega-3 fatty acids (0.06–0.10%).

The comparison between traditional sunflower oils and high-oleic sunflower oils underscored substantial differences in their fatty acid profiles. High-oleic sunflower oils predominantly comprised OA, making up 81.30–83.65% of the total fatty acids, in accordance with the regulations of the Food and Agriculture Organization. These regulations stipulate that high-oleic sunflower oils must contain not less than 70% OA as a percentage of total fatty acids [50]. Consequently, these oils exhibited the highest MUFA content among the oils analyzed, alongside low levels of SFAs and LA.

The fatty acid composition of olive oil exhibited a similarity to that of high-oleic sunflower oil, characterized by approximately 75% OA content. However, extra virgin olive oil, recognized for its superior quality and minimal processing, surpassed high-oleic sunflower oil in omega-3 fatty acid content. Extra virgin olive oil ranked second in OA and MUFA contents among the studied plant-based oils, resembling high-oleic sunflower oil in having a low LA content. The high content of OA and the relatively low levels of SFA in olive oil enhance its potential health-promoting properties.

Rapeseed oil featured notable levels of OA (60.55%) but to a lesser extent compared to high-oleic sunflower oil and olive oil. It exhibited the highest ALA content (7.60%) and the lowest total SFA content (7.17%) among the oils examined, with MUFA and PUFAs constituting over 90% of its total fatty acid content. Additionally, its favorable omega-6/omega-3 ratio (~2.8) and low SFA content underscored its nutritional potential.

Rapeseed oil serves as a constituent in various vegetable oil blends marketed as omega-3-, omega-6-, or omega-9-rich oils. For instance, FO36, labeled as an omega-3,6 vegetable oil, comprises 60% rapeseed oil and 40% sunflower oil, while FO9, marketed as an omega-9 vegetable oil, consists of 70% rapeseed oil and 30% high-oleic sunflower oil. Another alternative plant-based oil blend (COO3), also promoted for its omega-3 content, is formulated from a combination of five vegetable oils. These oil blends, characterized by low SFA content and abundant MUFAs, along with a relatively high PUFA content, highlight their potential in enhancing dietary health.

By analyzing the variations in fatty acid profiles post heating, significant insights can be obtained to guide dietary choices and cooking techniques, ultimately promoting optimal health and well-being. This comprehensive understanding aids in making informed decisions regarding food consumption and preparation methods, contributing to overall health promotion and maintenance.

In all analyzed oils, the levels of major SFAs, including palmitic and stearic acid increased with increases in the temperature and duration of treatment, ranging from 7.17–20.66% to 8.84–22.53% following 360 min heat exposure at 230 °C. Notably, in the case of olive oil and high-oleic sunflower oil, the observed increase was comparatively lower than that observed in refined sunflower oils. The increase in palmitic and stearic acid levels may be attributed to the degradation of double and triple bonds found in unsaturated fatty acids (UFAs), including mono-, di-, and polyunsaturated varieties. The cleavage of these bonds could subsequently facilitate the transformation of fatty acids into forms with either identical carbon counts or shorter chain lengths, resulting in the formation of SFAs, such as palmitic and stearic acids.

During heating, the OA content in sunflower, rapeseed, olive oils, and oil blends undergoes alterations due to various chemical reactions. The concentration of OA remained relatively stable during heat treatments at temperatures of 180 °C and 200 °C. High-oleic sunflower oil and olive oil, recognized for their abundant OA content and antioxidant compounds such as phenolics and tocopherols, exhibited minimal changes in OA during heating. Similarly, rapeseed oil and oil blends, which also contain substantial levels of OA, experienced modest increases, ranging from 9.97% to 25.30%. The observed increase in OA content can be attributed to the more rapid degradation of PUFAs, such as LA, during heating. The more PUFAs degrade, the more the relative proportion of OA in the oil increases due to selective degradation, resulting in a higher concentration of the relatively stable OA remaining. For sunflower oil, the OA level ranged from 26.18% to 40.17% before heating and increased to 36.34% to 53.38% after 360 min at 230 °C, indicating the most significant changes in OA among the oils studied and potentially suggesting the highest degradation of PUFAs.

**Figure 1:**
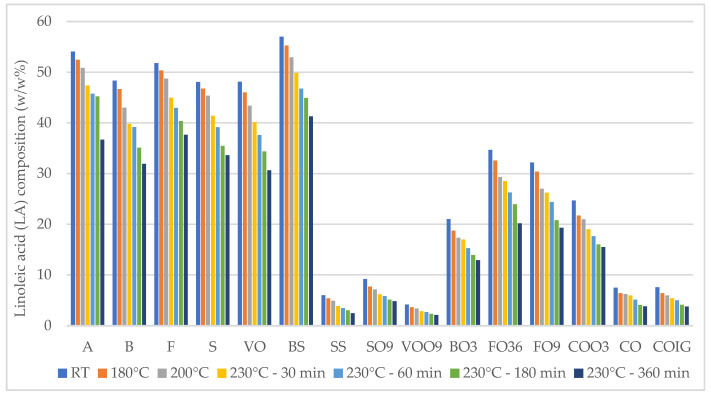
Changes in linoleic acid (LA) values in fifteen plant-based oils in fresh samples and at 180 °C, 200 °C, 230 °C—30 min, 60 min, 180 min, and 360 min.

After heating, the PUFA proportion was slightly reduced in all oils, indicating their degradation during the process being, among others, the precursor of some volatiles. The LA contents of the fifteen plant-based oils investigated across a temperature range from unheated to 230 °C are summarized in Figure 1. A consistent decline in LA content was observed in each oil following the heating periods. Sunflower oil exhibited the highest LA values in its fresh form, with a notable decrease observed in each subsequent sample during the heating process, demonstrating significant reductions compared to preceding samples. Specifically, in sunflower oils, the reduction in LA at 230 °C—360 min compared to unheated samples ranged from 27% to 36%. Blends of sunflower and rapeseed oils, which possessed the second-highest LA values, also experienced a significant reduction (40.06% to 41.75%) in LA content across all samples compared to fresh oil. Following the 230 °C—360 min heating phase, there was a substantial reduction in the LA content of olive oil and high-oleic sunflower oil, compared to the fresh sample, ranging from 49.27% to 50.19% and from 47.43% to 49.69%, respectively. This represents the most significant decrease in LA observed among all analyzed vegetable oils, despite their lower initial LA levels in their fresh form. The decrease in LA could be explained by the oxidation of UFA, which changes to primary and secondary oxidation products during the frying process.

**Figure 2:**
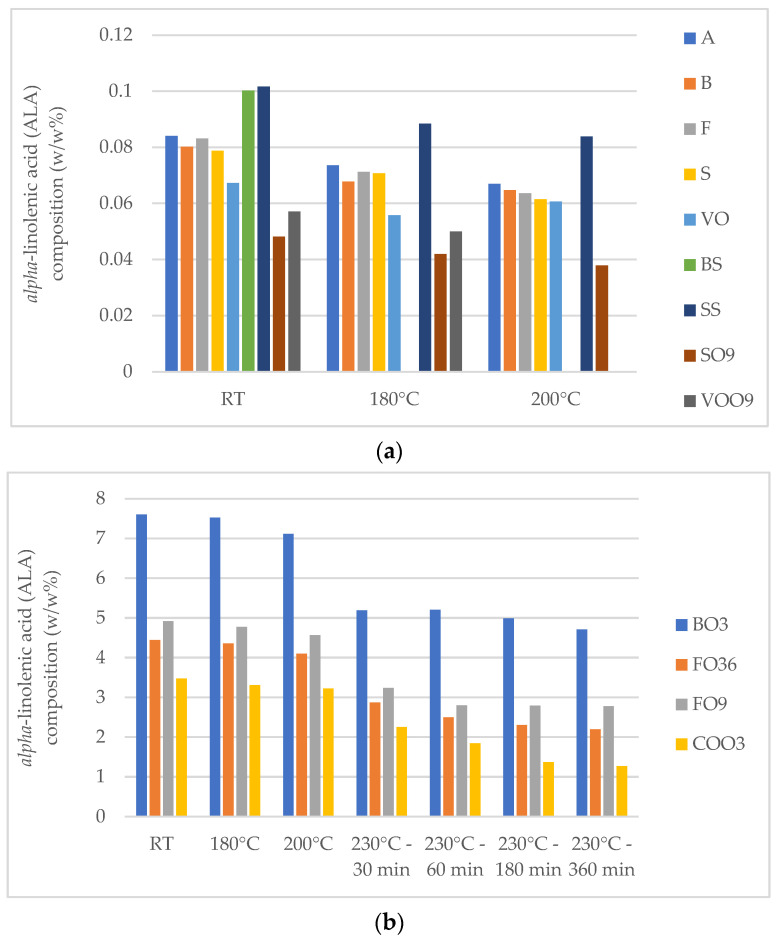
Changes in *alpha*-linolenic acid (ALA) values in (**a**) sunflower and high-oleic sunflower oils in fresh samples and at 180 °C and 200 °C; and (**b**) rapeseed oil and oil blends in fresh samples and at 180 °C, 200 °C, and 230 °C—30 min, 60 min, 180 min, and 360 min.

The ALA content of rapeseed oil, which exhibited the highest levels, as well as the blend of rapeseed and high-oleic sunflower oil, which ranked second in ALA values, demonstrated a significant decrease in each sample during the heating process compared to all preceding samples (Figure 2). Following heating at 230 °C, the reductions in ALA content compared to the unheated samples were 37.99% and 43.43–50.64%, respectively. This suggests that while rapeseed oil is omega-3-rich, it may not be the best option for high-temperature cooking if the goal is to preserve these sensitive fatty acids.

Significant decreases in ALA content were also noted in extra virgin olive oil at 180 °C and 200 °C compared to unheated samples, with reductions ranging from 8.82% to 13.02% compared to the fresh sample. Despite the initially minimal ALA content in high-oleic sunflower oil, heating at 230 °C resulted in ALA levels falling below the detection limit. Similarly, a significant decrease in ALA content was observed in sunflower oil between the unheated and 200 °C samples, with ALA undetectable in samples heated to 230 °C. Notably, unrefined sunflower oil (BS) exhibited an exception, with ALA undetectable at 180 °C. The significant decrease in ALA following heating at 230 °C indicates that sunflower oil exhibits greater sensitivity compared to other oils. This could suggest that sunflower oil contains fewer antioxidant compounds, such as tocopherols, than other oils, rendering it more susceptible to oxidation.

Therefore, vegetable oils abundant in MUFAs and PUFAs—known for their physiological benefits—undergo greater degradation during heating compared to fats containing predominantly SFAs. Consequently, UFAs exhibit reduced stability at elevated temperatures due to the susceptibility of their cis double bonds to saturation, isomerization into *trans* configuration, or other oxidative processes.

**Figure 3:**
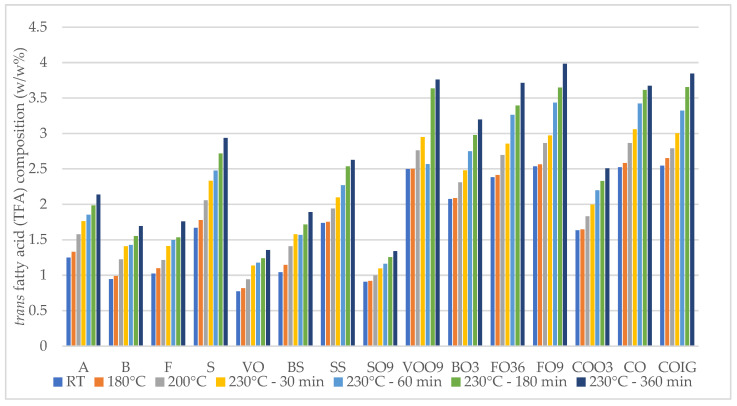
Changes in *trans* fatty acid (TFA) values in fifteen plant-based oils in fresh samples and at 180 °C, 200 °C, and 230 °C—30 min, 60 min, 180 min, and 360 min.

Upon subjecting the vegetable oils to predetermined temperatures and durations of heating, the TFA values in each oil markedly increased compared to their respective fresh samples (Figure 3), with the most pronounced elevation observed in sunflower oil (81.39%). High temperature treatment can induce the formation of *trans* fats in oils via oxidation processes. The extent of increase between the fresh and 230 °C—360 min samples ranged from 53.99% in rapeseed oil, 45.61–51.03% in extra virgin olive oil, 71.08–81.39% in sunflower oil, 47.74–50.52% in high-oleic sunflower oil, and 53.49–57.18% in oil blends. For refined sunflower oils, *trans* fat production was more influenced by temperature than prolonged heat exposure. On the other hand, vegetable oils, such as EVOO, high-oleic sunflower oil, and rapeseed oil were more affected by prolonged heat exposure at 230 °C. However, under the examined conditions (<230 °C—360 min), the *trans* fat content in vegetable oils remained significantly lower than the levels found in other food products like margarine made from partially hydrogenated oils [51].

**Figure 4:**
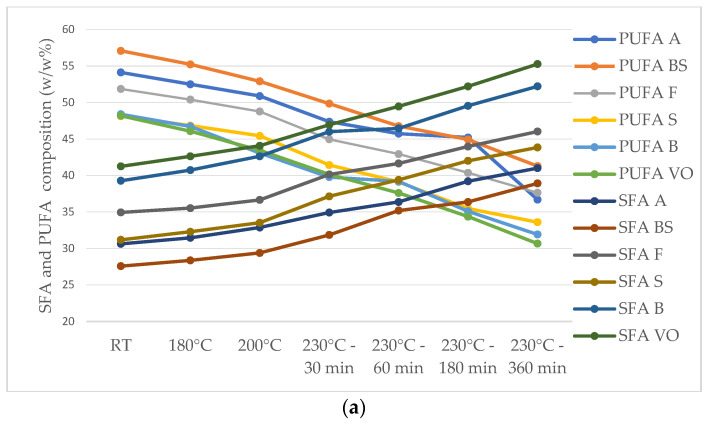

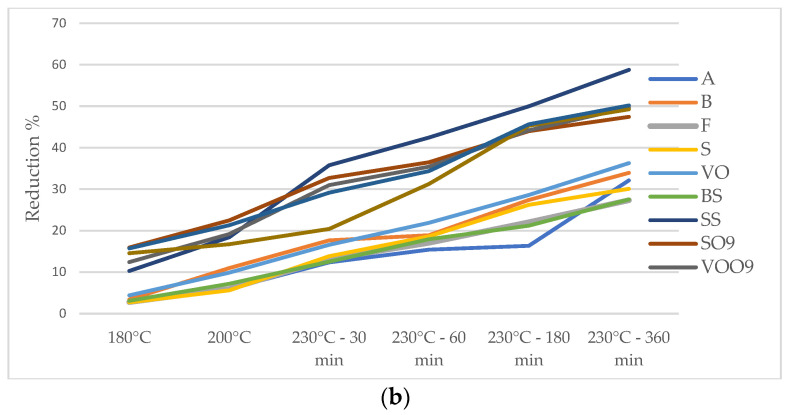
(**a**) SFA and PUFA changes during heating at 180 °C, 200 °C, and 230 °C—30 min, 60 min, 180 min, and 360 min—in sunflower oils. (**b**) LA reduction % in traditional and high-oleic sunflower oils.

According to Figure 4, heating sunflower oil led to an increase in the proportion of total SFAs and a decrease in total PUFAs (Figure 4a).

These changes were primarily driven by an elevation in palmitic acid, stearic acid, eicosanoic acid, behenic acid, and lignoceric acid levels, coupled with a decline in LA and ALA. While sunflower oil’s elevated SFA content provides it with oxidative stability, its significant PUFA content renders it prone to oxidation. Among the plant-based oils investigated, refined sunflower oils exhibited the most significant reductions in USFA content, ranging from 2.36% to 8.32%. This reduction consequently led to an increase in the total SFA content. Notably, refined sunflower oil exhibited the highest SFA content among the oils examined, and the heating process further augmented this content, which is considered undesirable from a health perspective. Sunflower oil is known as a valuable source of UFAs such as LA, an omega-6 fatty acid, and OA, an omega-9 fatty acid, but their concentration is affected by the thermal treatments. The relative OA content increased within a range of 12.46–17.45%, while its total PUFA content decreased by 12.48–17.79% after exposure to a temperature of 230 °C. Among sunflower oils, “VO” showed the most significant reduction in LA (36.30%) (Figure 4b). LA, in particular, undergoes oxidation and degradation during heating, forming aldehydes with roughly six carbon atoms, which could further polymerize to produce fatty acids with a carbon skeleton of twelve or more atoms [14].

High-oleic sunflower oils, known for their elevated OA content, showed the least modification in this fatty acid compared to all vegetable oils analyzed in this study. When compared to traditional sunflower oil, the total increase in high-oleic varieties was nearly tenfold smaller. A similar trend was observed in olive oil, where the increase ranged from 4.21% to 4.51%, ranking second in terms of minimal change. Therefore, after subjecting the oil to heating at 230 °C for 360 min, high-oleic sunflower oil demonstrated the highest MUFA content among the oils analyzed. While the reduction in ALA, an essential omega-3 fatty acid, was higher at 200 °C in high-oleic sunflower oil compared to olive oil, it remained lower than in traditional sunflower oil. Conversely, LA experienced increased reduction in both high-oleic sunflower oil and olive oil, ranging from 47.43% to 58.77% and 49.27% to 50.19%, respectively (Figure 4b). These findings suggest that OA plays a central role in the thermal stability of high-oleic sunflower oil and olive oil. This could be attributed to the presence of phenolic compounds and tocopherols in olive oil, which provide antioxidant properties and may contribute to its thermal stability during frying, despite its lower OA content compared to high-oleic sunflower oil [3].

The most frequent plant-based oil combinations found in the Romanian market comprised sunflower oil (either traditional or high-oleic) and rapeseed oil. These products are marketed as vegetable oils boasting a rich profile of omega-3, omega-6, or omega-9 fatty acids. The composition of these oil blends, along with rapeseed oil alone, demonstrates a noteworthy concentration of omega-3 fatty acids, underscoring their potential health benefits. Notably, during deep-fat frying processes, a consistent decline in ALA content was observed across these oils. BO3, a refined rapeseed oil, showed the most stability during the deep-fat frying process regarding ALA content. Blending high-oleic sunflower oil with rapeseed oil appears to enhance the stability of ALA in the resultant oil, as evidenced by the minimal reduction observed during frying, compared to traditional sunflower oil alone. The blends also aim to optimize the PUFA/SFA ratio, which is crucial for maintaining cardiovascular health. Vegetable oil mixtures can enhance the stability of these fatty acids during heating, suggesting a potential strategy for maintaining the nutritional quality of oils exposed to high temperatures. In their initial state, rapeseed oils exhibited the lowest total SFA content, whereas during heating, high-oleic sunflower oil displayed the lowest relative total SFA content. This alteration became prominent with extended heating durations rather than with elevated temperatures. The results suggest that the incorporation of sunflower oil into rapeseed oil blends could enhance their storage stability, attributable to the elevated levels of monoenoic fatty acids and low SFA content within the blends. Also, the initial levels of UFAs in vegetable oils were particularly elevated in omega-9-enriched variants, exemplified by rapeseed oils (92.82%), high-oleic sunflower oils (ranging from 90.80% to 91.80%), and oil blends (ranging from 89.97% to 91.80%). It is worth noting that oil blends exhibited a diminished reduction, suggesting that mixing oils helps mitigate significant decreases in UFA levels within oil formulations. Consequently, this enhances the stability of blended oils during deep-fat frying in comparison to unmixed oils.

Various indices, based on the fatty acid composition, are employed to elucidate the health implications of diverse dietary choices [4,52,53]. PUFA/SFA is the most commonly used index for assessing the nutritional value of foods. It serves as a prominent indicator for evaluating the cardiovascular health implications of dietary intake. SFAs, particularly C12:0, C14:0, and palmitic acid, are predominantly recognized as pro-atherogenic [4,53] and pro-thrombogenic, [53] whereas UFAs (including MUFAs, n-3, and n-6 PUFAs) exhibit contrasting properties, being anti-atherogenic and anti-thrombogenic. Consequently, a higher PUFA/SFA ratio correlates with more favorable effects on cardiovascular health. Additionally, the PUFA/SFA ratio serves as a reliable indicator for assessing oil oxidation. The frying temperature and duration, alongside the characteristics of the oils employed in this process, significantly influence the balance between these two types of fatty acids. In the present investigation, all examined oils displayed a notable decrease in the PUFA/SFA value following heating, indicative of less favorable health implications. In their unprocessed state, all vegetable oils examined in this study exceeded the recommended threshold of 0.40 for the PUFA/SFA ratio. However, when subjected to heating, the PUFA/SFA ratio decreased notably in olive oil, dropping to 0.2 after 360 min at 230 °C. This suggests that it is advisable to refrain from using olive oil for heating purposes beyond temperatures of 200 °C. The USFA/SFA ratio in vegetable oils in the modern diet is currently a subject of considerable interest. This correlation serves as a reliable metric, with prior research suggesting that the recommended ratio should surpass 1.6. Even after exposing the oils to 230 °C for various durations, this ratio did not drop below 3 in any of the oils. Nonetheless, heating led to a decrease in USFA/SFA ratio across all examined vegetable oils.

To assess alterations in fatty acid composition during frying, the ratios of USFA/palmitic acid were also examined, as detailed in Table 3. This ratio serves as a valuable indicator for tracking changes in fatty acid composition under thermal stresses. As frying duration extended, the UFA-to-palmitic acid ratios notably declined, particularly at elevated temperatures. This suggests that frying at higher temperatures may accelerate the degradation of PUFAs.

The percentage reductions in the LA/palmitic acid ratio varied across the different oils studied: refined sunflower oil, high-oleic sunflower oil, rapeseed oil, oil blends, and olive oil exhibited reductions ranging from 34.88% to 47.47%, 48.81% to 53.00%, 59.88%, 49.79% to 59.48%, and 50.83% to 51.87%, respectively. Notably, unmixed rapeseed oil experienced a significant decrease in the LA/palmitic acid ratio compared to other oils, whereas blending it with other oils resulted in a less pronounced reduction in the ratio than observed in the individual oil. Thus, the LA/palmitic acid ratio was utilized as an indicator of the extent of oxidative deterioration in the frying oil samples.

Health authorities and professional bodies have issued guidance aiming to decrease the ratio of n-6 to n-3 PUFAs in the diet. This ratio has a strong association with mortality linked to cancer, cardiovascular diseases, inflammatory conditions, and autoimmune disorders. It has been reported that the primary risk factor for arteriosclerosis and ischemic heart disease is not hypercholesterolemia or high cholesterol consumption, but rather a high n-6/n-3 PUFA ratio. Currently, in most industrialized Western societies, this ratio stands at approximately 15–20:1, whereas nutritional experts advocate for an n-6/n-3 ratio of less than 4:1 [54]. In general, vegetable oils with lower LA/ALA ratios are considered more balanced in terms of omega-6 to omega-3 fatty acid intake, a characteristic often recommended for promoting a healthier dietary profile [52]. In the case of refined sunflower oil, the n-6/ n-3 PUFA ratios were notably higher at each investigated time point compared to the preceding ones, highlighting potential concerns regarding their impact on dietary health. For rapeseed oils and oil blends, the n-6/n-3 ratio showed a significant increase only after heating at 230 °C for 60 min. Similarly, in olive oil, this ratio did not significantly change during low heating temperatures, resembling the stability observed in rapeseed oils, suggesting potential benefits in maintaining a balanced fatty acid profile under various cooking conditions.

### 3.2. Multivariate Data Analysis

The compositional changes in various oils subjected to different temperatures and exposure times were also investigated by multivariate data analysis (MVDA) aiming to understand how the chemical composition of oils varies under these conditions. By employing unsupervised principal component analysis (PCA) models, the complexity of the high-dimensionality chromatographic data was reduced to elucidate patterns and correlations within the data, focusing on the conditions presented in Table 4.

**Figure 5:**
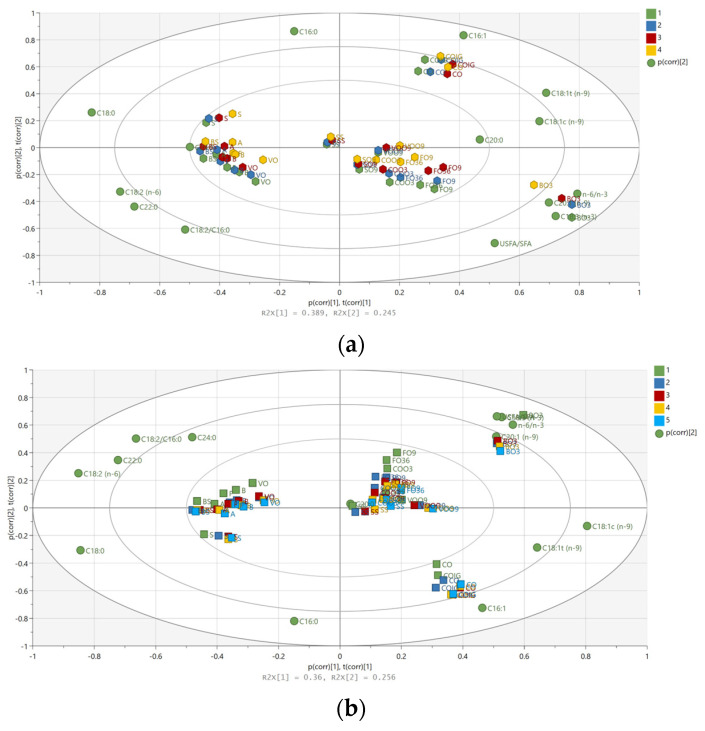
Biplot of PCA-X model (PC1 vs. PC2) of the analyzed edible oil samples, reflecting their fatty acid composition (**a**) at RT compared to after heating up to 230 °C, colored according to classes (green—RT; blue—180 °C; red—200 °C; yellow—230 °C); and (**b**) at different heating times at 230 °C, colored according to classes (green—RT; dark blue—30 min at 230 °C; red—60 min at 230 °C; yellow—180 min at 230 °C; blue—360 min at 230 °C).

For a better overview and easier data interpretation, the scores and loadings of the PCA model were co-charted in biplots (Figure 5a,b). Models with four principal components (PCs) emerged which explained >90% of the cumulative variance. The biplot of the first two PCs, accounting for more than 60% of the total variability in the analyzed dataset, seemed to group and order the edible oil samples according to the type of oil and frying time and temperatures.

**Figure 6:**
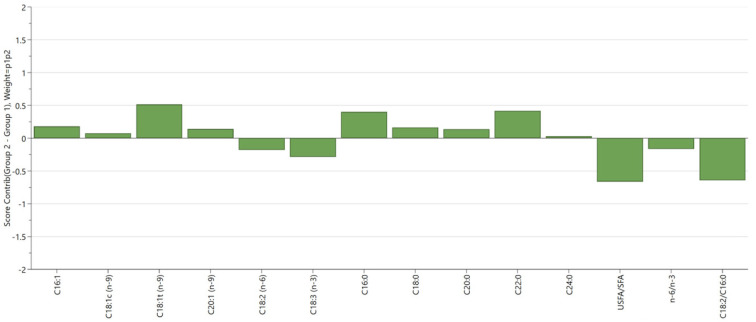
Score contributions after 30 min at 230 °C vs. control (RT).

The PCA biplot (PC1 vs. PC2) indicates the distinct clustering of oil compositions between RT and 180 °C, 200 °C, and 230 °C, demonstrating the impact of increasing temperature (Figure 5a). Higher temperatures (200 °C and 230 °C) result in more significant compositional changes compared to lower temperatures (RT and 180 °C). The PCA (Figure 6) shows that the oil composition at 230 °C is markedly different from that at RT, with a significant increase in SFAs (i.e., palmitic acid, stearic acid) in the heated traditional sunflower oils. The heated oil blends and the high-oleic sunflower oils, VOO9 and SO9, gravitate around the origin of the biplot, indicating a higher stability against extreme heat exposures due to a more balanced composition in terms of saturated and polyunsaturated fatty acids.

Time-dependent monitoring (up to 360 min) of the studied oils shows that significant changes in their composition could already be detected even after 30 min of exposure, with noticeable shifts along PC1 in the PCA plot (Figure 5b), which become even more evident by extending the heat treatment. Prolonged exposure times lead to progressive changes in omega-3 edible oil composition, evidenced by a decline in linoleic acid content and the USFA/SFA ratio in comparison with the control (RT) group. Both traditional and high-oleic sunflower oil types showed substantial compositional shifts after prolonged exposure, with distinct groupings in the PCA plots. Sunflower oils (A, B, BS, F, S, SS, VO) and omega-9 oils (SO9, VOO9, FO9) display different trajectories of compositional change, indicating varying stability and reactivity, with sunflower oils showing more significant variations in FA content (i.e., OA) over time. Omega-3 and -6 oils demonstrate distinct chemical transformations compared to omega-9 oils, suggesting different mechanisms of degradation or stability under thermal stress. Omega-3- and omega-6-enriched vegetable oils show greater changes after exposure to high temperatures, with more a significant degradation of PUFA compared to oils labeled as omega-9. However, both types of oils trend towards higher levels of SFA, OA, and trans-9-elaidic acid.

MVDA provided valuable insights into the compositional dynamics of oils under varying thermal conditions. For culinary applications involving high-temperature cooking (above 200 °C), it is advisable to use oils with a more balanced composition of saturated and unsaturated fatty acids, such as high-oleic sunflower oils or omega-9-enriched oils. These oils exhibit higher stability and lower rates of degradation under thermal stress. To maintain the nutritional quality of omega-3- and omega-6-enriched oils, it is essential to minimize their exposure time to high temperatures. Even at lower temperatures, prolonged heating should be avoided to prevent significant compositional changes and the loss of beneficial PUFAs.

The findings highlight the importance of temperature and exposure time in determining the chemical stability of different oil types, with significant implications for their use in culinary and industrial applications.

## 4. Conclusions

This research underscores the significance role of fatty acid composition in influencing the thermal stability of oils. Upon subjecting vegetable oils to varying temperatures and durations of heating, a notable decline in the levels of PUFAs, which are predominantly beneficial from a nutritional standpoint, was observed, accompanied by significant increases in *trans* isomers and SFA, known for their less favorable nutritional effects. These results suggest that during frying, the loss of PUFAs can be attributed partly to *cis*-*trans* isomerization and partly to the saturation of double bonds within the fatty acid chains. The type of oil employed significantly influenced these changes in nutritional indices.

The increasing consumer demand for healthier cooking oils has led to innovations in oil processing and blending. The practice of blending different types of oils to achieve an optimal balance of thermal stability and nutritional value is particularly promising. Oil blends comprising rapeseed and high-oleic sunflower oils demonstrated the most stability at high temperatures, reducing the formation of harmful compounds and exhibiting minimal alterations in nutritional index values. On the other hand, sunflower oils proved to be the most susceptible to oxidation, due to their high LA content, experiencing the greatest changes in their fatty acid composition. The findings suggest that blending high-oleic sunflower oil with other vegetable oils can enhance thermal stability, preserving the nutritional quality of the oil during frying. This strategy can mitigate the loss of essential fatty acids and maintain a favorable PUFA/SFA ratio, crucial for cardiovascular health. These findings reinforce the recommendation to opt for oils rich in PUFAs and low in SFAs for food preparation, while advocating for frying at lower temperatures and avoiding multiple reheating cycles. Additionally, they support broader public health directives advising against the prolonged or repeated use of cooking oils, particularly pertinent in informal food sectors in low- and middle-income countries where such practices may be prevalent.

Marketing omega-fortified vegetable oils, therefore, requires a careful balance. The health claims associated with these oils, such as being heart-healthy or cholesterol-free, are compelling sales points. Packaging that emphasizes these attributes can effectively attract health-conscious consumers. However, the marketing strategies must also align with the scientific realities these products face during cooking. It is essential that the marketing of these oils remains honest about the conditions under which their health benefits can be fully realized, such as recommending lower cooking temperatures to maintain fatty acid integrity.

In conclusion, omega-fortified vegetable oils represent a significant advancement in dietary fats that cater to health-conscious individuals. However, their thermal instability at high frying temperatures poses a challenge. This necessitates continued research and technological innovation to develop more stable oil varieties without compromising their nutritional benefits. As the market for these products grows, regulatory oversight will also be crucial to ensure that health claims are substantiated, guiding consumers towards safer and healthier dietary choices.

## Figures and Tables

**Table 1:** Characteristics of vegetable oil samples used for GC-MS analysis.

Oil ID	Composition	Brand	Abbreviation
A	Refined sunflower oil	Brand 6	RS
BS	Unrefined sunflower oil (extra virgin)	Brand 7	URS
BO3	Refined rapeseed oil	Brand 1	RR
CO	Extra virgin olive oil	Brand 3	EVOO
COO3	Oil blend (sunflower, grapeseed, corn, flaxseed, rice bran)	Brand 3	MX
FO36	Refined rapeseed oil (60%) and sunflower oil (40%)	Brand 2	MX
F	Refined sunflower oil	Brand 2	RS
COIG	Extra virgin olive oil	Brand 3	EVOO
SS	Unrefined sunflower oil (extra virgin)	Brand 8	URS
S	Refined sunflower oil	Brand 4	RS
SO9	Refined high-oleic sunflower oil	Brand 4	RS
B	Refined sunflower oil	Brand 1	RS
FO9	Refined rapeseed oil (70%) and refined sunflower oil (high-oleic)	Brand 2	MX
VO	Refined sunflower oil	Brand 5	RS
VOO9	Refined high-oleic sunflower oil	Brand 5	RS

**Table 2:** Fatty acid composition of vegetable oils in unheated samples, as w/w% of total fatty acids.

Vegetable Oils/Fatty Acids	Rapeseed Oil (%)	Sunflower Oil (%)	High-Oleic Sunflower Oil (%)	EVOO (%)	Oil Blends (%)
saturated fatty acid (SFA)					
C16:0	4.26	5.89–12.38	4.09–4.33	12.56–14.77	5.03–5.74
C18:0	1.48	3.12–6.63	2.50–2.84	2.98–3.16	2.55–2.78
C20:0	0.62	0.28–0.36	0.26–0.33	0.49–0.52	0.30–0.58
C22:0	0.48	0.91–1.10	0.94–1.20	0.23–0.24	0.74–0.80
C24:0	0.29	0.37–0.49	0.37–0.48	0.54–0.59	0.34–0.38
SFA TOTAL	7.17	10.53–20.66	8.19–9.19	16.86–19.24	8.98–10.02
monounsaturated fatty acid (MUFA)					
C16:1	0.27	0.14–0.18	0.15–0. 17	1.15–1.28	0.15–0.24
C18:1c (n-9)	60.55	26.18–40.17	81.30–83.65	68.26–71.08	47.43–60.79
C18:1t (n-9)	2.07	0.77–1.66	0.90–2. 49	2.52–2.54	1.63–2.53
C20:1 (n-9)	1.30	0.16–0.20	0.20–0.26	0.26–0.35	0.24–0.90
MUFA TOTAL	64.20	27.58–41.25	82.58–86.60	72.44–75.02	50.87–62.83
polyunsaturated fatty acid (PUFA)					
C18:2 (n-6)	21.02	48.07–56.99	4.14–9.17	7.46–7.57	24.70–34.65
C18:3 (n-3)	7.60	0.06–0.10	0.04–0.05	0.64–0.73	3.47–4.91
PUFA TOTAL	28.62	48.15–57.09	4.19–9.22	8.10–8.30	28.18–39.09

**Table 3:** Ratios between palmitic acid and unsaturated fatty acids of the oils before and during heating the vegetable oils.

Vegetable Oil Type	Vegetable Oil ID	RT	180 °C	200 °C	30 min @230 °C	60 min @230 °C	180 min @230 °C	360 min @230 °C
Sunflower oil	A	9.44	8.95	8.83	8.62	8.50	8.60	7.71
	B	12.88	12.83	11.85	12.00	11.77	11.05	10.78
	F	11.75	11.10	10.62	10.38	10.19	9.99	9.74
	S	6.41	6.34	6.41	6.25	6.12	5.80	5.83
	VO	15.18	14.76	13.18	12.37	12.64	12.21	12.03
	BS	9.77	9.00	8.48	8.25	8.48	8.15	7.62
	SS	15.36	15.19	15.00	14.76	15.67	16.91	19.45
High-oleic sunflower oil	SO9	22.40	20.47	20.79	19.19	19.13	19.50	19.81
	VOO9	20.96	20.99	20.80	20.25	20.43	20.75	21.73
Rapeseed oil	BO3	21.77	17.55	17.21	16.52	16.23	14.13	13.65
Oil blend	FO36	15.66	14.44	14.09	13.16	13.24	12.90	12.20
	FO9	17.55	15.31	13.24	12.30	12.77	12.13	11.28
	COO3	18.06	16.54	16.19	15.34	15.15	14.62	14.01
EVOO	CO	6.61	6.52	6.54	6.30	6.24	6.21	6.24
	COIG	5.47	5.35	5.63	5.37	5.56	5.43	5.36

**Table 4:** MVDA variables.

Temperature Variations	Exposure Time at 230 °C	Vegetable Oil Types
Room Temperature (RT)	30 min	Sunflower oil (A, B, BS, F, S, SS, VO)
180 °C	60 min	Omega-9 oil (FO9, SO9, VOO9)
200 °C	180 min	Omega-3, 6 oil (BO3, COO3, FO36)
230 °C	360 min	Olive oil (CO, COIG)

## Data Availability

The original contributions presented in the study are included in the article. Further inquiries can be directed to the corresponding author.
